# High-Performance Voltammetric Aptasensing Platform for Ultrasensitive Detection of Bisphenol A as an Environmental Pollutant

**DOI:** 10.3389/fbioe.2020.574846

**Published:** 2020-09-04

**Authors:** Shokoufeh Hassani, Milad Rezaei Akmal, Armin Salek Maghsoudi, Soheila Rahmani, Faezeh Vakhshiteh, Parviz Norouzi, Mohammad Reza Ganjali, Mohammad Abdollahi

**Affiliations:** ^1^Toxicology and Diseases Group (TDG), Pharmaceutical Sciences Research Center (PSRC), The Institute of Pharmaceutical Sciences (TIPS), and Department of Toxicology and Pharmacology, School of Pharmacy, Tehran University of Medical Sciences, Tehran, Iran; ^2^Center of Excellence in Electrochemistry, Faculty of Chemistry, University of Tehran, Tehran, Iran; ^3^Nanotechnology Research Centre, School of Pharmacy, Tehran University of Medical Sciences, Tehran, Iran; ^4^Endocrinology and Metabolism Molecular-Cellular Sciences Institute, Tehran University of Medical Sciences, Tehran, Iran

**Keywords:** bisphenol A, endocrine-disrupting compounds, electrochemical aptasensor, environmental pollutant, toxicology

## Abstract

Bisphenol A (BPA) as a pervasive endocrine-disrupting compound (EDC) has been shown to cause multiple detrimental effects including cardiovascular disorders, pregnancy complications, obesity, glucose metabolism disorders, and reproductive toxicity even at a concentration as low as tolerable daily intake (TDI) (4 μg/kg/day). In the present study, a novel ultra-sensitive, electrochemical aptasensor was designed using a screen-printed carbon electrode (SPCE) modified by gold nanoparticles (Au NPs) conjugated to thiolated aptamers for accurate determination of BPA in biological, industrial and environmental samples. To characterize the electrochemical properties of the aptasensor, cyclic voltammetry (CV) and electrochemical impedance spectroscopy (EIS) were implemented. Detection of BPA was also performed through differential pulse voltammetry (DPV) in [Fe(CN)_6_]^3–/4–^ electrolyte solution. Under optimum condition, the present electrochemical aptasensor demonstrated an outstanding linear response in the concentration range of 1 pM to 10 nM with a remarkably low limit of detection of 0.113 pM. Due to the superb affinity between anti-BPA aptamers and BPA molecules, the designed aptasensor did not show any significant interaction with other analytes in real samples. Also, fabricated biosensor remained perfectly stable in long-term storage. The analytical results of the fabricated aptasensor are well compatible with those obtained by the ELISA method, indicating the trustworthiness and reasonable accuracy of the application of aptasensor in real samples. Overall, the proposed aptasensor would be a credible and economical method of precise, reproducible, and highly selective detection of minimum levels of BPA in food containers and clinical samples. This would be a promising strategy to enhance the safety of food products and reduce the risk of BPA daily exposure.

## Introduction

Endocrine-disrupting compounds (EDCs) are among the most widespread industrial chemicals used as plasticizers, pesticides, and storage containers (Alonso-Magdalena et al., 2011; Rahmani et al., 2018). The environmental persistence of EDCs is bound to have detrimental effects on the ecosystem and human health (Alonso-Magdalena et al., 2006). One of the EDCs having been vigorously focused on in recent years is bisphenol A [2, 2-bis (4-hydroxyphenyl) propane, BPA], which is a synthetic estrogen applied in polycarbonate polymers and epoxy resins (Rajabnejad et al., 2020). Due to its chemical resistance and hardness, BPA is mainly used in the manufacture of plastics found in several consumer goods, including baby bottles, food containers, toys, mobile phones, hospital and laboratory equipment, and laptops (Kang et al., 2006; Salek-Maghsoudi et al., 2019). As a weak estrogenic compound, BPA interferes in multiple cellular pathways of apoptosis, proliferation, and migration (Ma et al., 2019; Nomiri et al., 2019; Rahmani et al., 2020). BPA has been shown to cause numerous damaging health effects, including cardiovascular disorders, pregnancy complications, obesity, glucose metabolism disorders and reproductive toxicity (Kabir et al., 2015; Ejaredar et al., 2017; Salek-Maghsoudi et al., 2018; Rajabnejad et al., 2020) even at a concentration as low as tolerable daily intake (TDI) (4 μg/kg/day) (Zehani et al., 2015; Erjavec et al., 2016). Although various techniques have already been developed, more cost-effective, robust, and sensitive detection methods have yet to be designed to monitor the level of hazardous chemicals in food and environmental samples (Hassani et al., 2017; Baghayeri et al., 2018). At the moment, BPA is determined via different types of analytical methods such as liquid chromatography coupled with mass spectrometry (LC-MS), high-performance liquid chromatography (HPLC) (Hassani et al., 2013), capillary electrophoresis (CE) (Zhang et al., 2015), gas chromatography coupled with mass spectrometry (GC-MS) (Cao et al., 2015), and enzyme-linked immunosorbent assay (ELISA) (Hassani et al., 2018b). Despite having many benefits, these techniques suffer from some noticeable demerits such as high-priced chemicals, painstaking analysis procedure and the necessity of labeling antibodies with enzymatic materials, which make them less suitable for routine measurements (Vílchez et al., 2001; García and Losada, 2004; Abnous et al., 2018). Therefore, the implementation of substitute methods with such high selectivity and sensitivity, such as aptamers, can be of paramount importance, especially in case of environmental contaminants whose long-term effects on wildlife and human health can be devastating (Hassani et al., 2018a). Having prominent advantages including unique stability in extreme conditions of pH and temperature, user-friendly pretreatment, straightforward immobilization, small size and portable platforms, aptamers have become superior to antibody-based assays (Mehrotra, 2016). Aptamers are single-stranded DNAs or RNAs selected by SELEX technology (Systematic Evolution of Ligands by Exponential Enrichment) and can be assembled straightforwardly and are easily accessible (Röthlisberger and Hollenstein, 2018; Villalonga et al., 2020). Additionally, their high specificity and vigorous affinity toward corresponding targets, aptamers can be effortlessly modified through the incorporation of a wide range of functional groups, making them perfectly suitable for a variety of applications (Chen and Yang, 2015; Derikvandi et al., 2016; Ye et al., 2017). Generally, biosensing detection platforms with their substantial specificity and cost-effectiveness offer reliable substitutes to the conventional analytical assays. A biosensor is typically composed of the biological sensing element and a transducer, which is divided into three categories: electrochemical (Benvidi et al., 2018), optical (Qi et al., 2019), and physical (Vigneshvar et al., 2016; Zahed et al., 2020). Screen-printed carbon electrodes (SPCEs) applied in electrochemical (EC) biosensors offer an enormous wealth of advantages, including exceptional sensitivity, miniaturization capacity, relatively low cost, less time-consuming detection process and reproducible results with a small volume of sample needed for each measurement and feasibility for large scales of production (Wan et al., 2015; Zhang et al., 2016). Moreover, modification of the surface of SPEs with nanomaterials (NMs), particularly gold nanoparticles (AuNPs), can considerably enhance their performance (Roushani and Shahdost-fard, 2015; Benvidi et al., 2016). Herein, we report on the fabrication of a novel signal-off electrochemical aptasensor based on anti-BPA aptamer conjugated AuNP-modified SPCE, which can offer precise, reproducible, and extremely sensitive detection of minimum levels of BPA in real samples.

## Materials and Methods

### Experimental Animals

Wistar male rats (200–250 g, 2.5-month old) were obtained from the animal house of the Tehran University of Medical Sciences (TUMS). They were stored at room temperature (22 ± 2°C), humidity (55 ± 5%), and a 12:12 h light-dark cycle and had free access to food and water. The study was performed according to ethical guidelines on the use of animals in research, and the protocol was approved by the Institute of Pharmaceutical Sciences (TIPS) Ethics Committee under code number IR.TUMS.VCR.REC.1398.328.

### Materials

Gold chloride trihydrate (HAuCl_4_.3H_2_O), phosphate-buffered saline (PBS), H_2_SO_4_, NaCl, KCl, NaH_2_PO_4_, Na_2_HPO_4_, MgCl_2_, Tris (2-carboxyethyl) phosphine hydrochloride (TCEP), 6-mercapto-1-hexanol (MCH), potassium ferricyanide and ferrocyanide were purchased from (Sigma-Aldrich, St. Louis, MO, United States). BPA, bisphenol B (BPB), hexafluorobisphenol A (6F-BPA), hydrogenated bisphenol A (HBPA), and estradiol were supplied from Merck (Darmstadt, Germany). Thiolated BPA aptamer with the following sequences based on previous studies (Baghayeri et al., 2018) (5′-Thiol-(CH_2_)_6_-CCGGTGGGTGG TCAGGTGGGATAGCGTTCCGCGTATGGCCCAGCGCATCA CGGGTTCGCACCA-3′) was synthesized by MWG-BIOTECH, Germany. Ultra-pure water with a resistance of 18MΩ.cm (EMD Millipore, Billerica, MA, United States) was used to prepare all solutions. The stock solution of aptamer (20 μM) was prepared in the PBS (0.1 M, pH 7.5) and stored at −20°C.

### Apparatus

To perform the electrochemical measurements, an AUTOLAB PGSTAT 101 potentiostat/galvanostat (Metrohm Autolab BV, Utrecht, Switzerland) supporting NOVA 2.1 software was used. All the electrochemical techniques employed for the SPCE characterization were performed in 0.1 M PBS (pH 7.5) containing [Fe(CN)_6_]^3–/4–^/KCl (5 mM/0.1 M) electrolyte solution as a redox probe using three distinct EC techniques cyclic voltammetry (CV), electrochemical impedance spectroscopy (EIS), and differential pulse voltammetry (DPV). EIS was carried out through an AC (alternating current) voltage amplitude modulation of +10 mV with frequency ranging between 100 mHz and 100 kHz. CV technique was conducted in order to assess the procedure of fabrication of the aptasensor. The potential was calculated in the range between −0.4 and +0.7 V at a scan rate of 0.1 V/s. DPV method was used under experimental conditions of potential ranging from +0.5 to −0.3 V, and amplitude modulation of 0.05 V. Following electrodeposition procedure, the morphology of AuNPs was thoroughly assessed by FESEM (Hitachi S-4160, Japan). SPCEs with a reference number of DRP-110 were purchased from (DropSens, Spain).

### AuNPs-Modified SPCEs Assembly

At first, the activation process was performed in 0.5 M H_2_SO_4_ solution with CV scanning from −0.1 to +1.3 V and a scan rate of 0.1 V/s until steady voltammograms were acquired. Before the activation, the SPCE was characterized via CV measurements in the range between −0.4 and +0.7 V in 0.1 M PBS (pH 7.4) containing [Fe(CN)_6_]^3–/4–^/KCl (5 mM/0.1 M) electrolyte solution as the electrochemical redox probe with a scan rate of 0.1 V/s. Surface modification of the SPCEs by AuNPs was based on a previously described procedure (Charoenkitamorn et al., 2015). An electrodeposition potential of −0.5 V was applied in 5 mM HAuCl_4_ in 0.5 M H_2_SO_4_ solution with 200 s as an optimized deposition time, following the activation of the electrode surface. The efficiency of electrodeposition was assessed through CV measurements, and then the voltammograms of AuNP-modified SPCE and bare-activated SPCE were compared. At last, AuNPs deposition on SPCEs was confirmed by using FESEM.

### Aptamer Immobilization Onto AuNPs/SPCEs Surface

To modify the electrode surface by immobilized bioreceptor, BPA aptamer (20 μM stock solution) was incubated with 10 mM TCEP solution, and the mixture was kept for 1 h at 25°C to reduce disulfide bonds. The stock solution of BPA aptamer was then diluted with 0.1 M PBS (pH 7.5) to 1 μM. Afterward, 10 μL of aptamer solution (1 μM) was added dropwise onto the AuNPs-modified SPCE and subsequently incubated at 4°C for 16 h in the dark, which resulted in the formation of self-assembled monolayers (SAM). Shortly after that, the modified electrode was washed thoroughly with ultrapure distilled water to remove remaining unattached aptamers from its surface. For the last step, the non-specific binding sites were blocked, adding 2 mM MCH solution. After 30 min incubation, modified SPCE was again rinsed with ultrapure distilled water, and the electrode was dried under a stream of N_2_ gas.

### Electrochemical Detection of BPA

**SCHEME 1:**
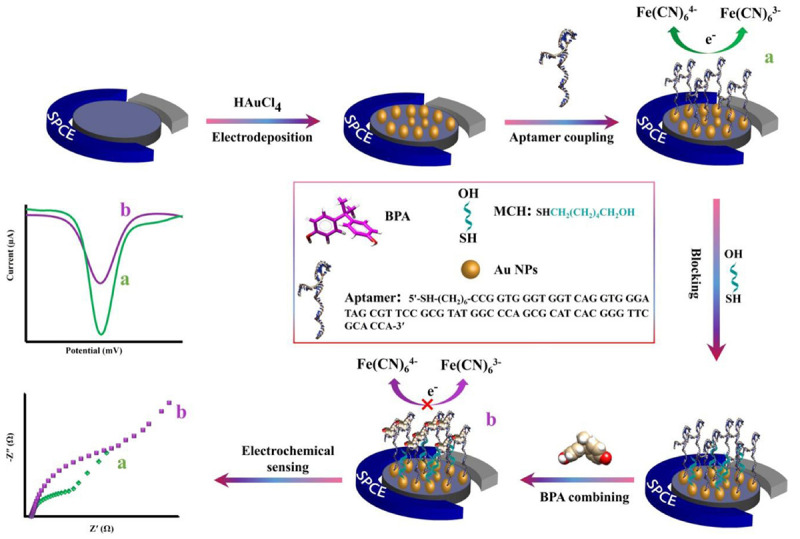
The schematic representation of the electrochemical aptasensor fabrication procedure for the detection of BPA.

After immobilizing aptamers on modified SCPE, different solutions of BPA concentrations were added on the modified electrodes (MCH/aptamer/AuNPs/SPCEs). After 40 min incubation of the aptasensor at 25°C, the electrodes were washed using ultrapure water and dried under a stream of N_2_ gas. The aptasensor was then placed into an electrochemical cell filled with 0.1 M PBS (pH 7.5) containing [Fe(CN)_6_]^3–/4–^/KCl (5 mM/0.1 M) electrolyte solution. The DPV measurements of BPA/MCH/thiolated aptamer/AuNPs/SPCEs were recorded in different concentrations of BPA under the experimental condition of potential ranging between +0.5 and −0.3 V, modulation time of 0.05 s and interval time of 0.2 s. The procedure of the aptasensors fabrication is illustrated in Scheme 1.

## Results and Discussion

### Characterization of the AuNPs

**FIGURE 1:**
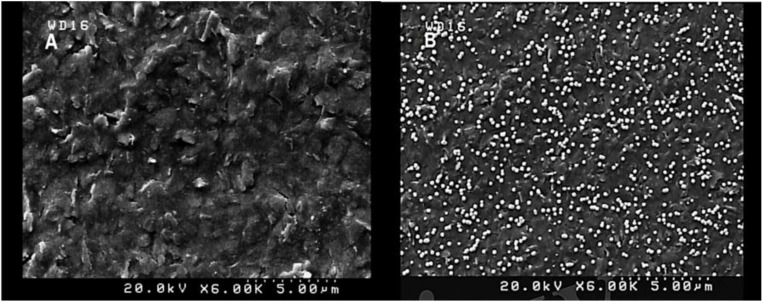
FESEM images of unmodified SPCE **(A)** and modified (AuNPs) SCPE **(B)** using electrodeposition method (5 mM HAuCl_4_ in 0.5 M H_2_SO_4_; time: 200 s).

Morphological characteristics of the bare SPCE and AuNPs modified SPCE was investigated by FESEM (Figure 1). Signal transduction was substantially enhanced by exploiting AuNPs, which successfully expanded the SPCE’s electroactive surface area and increased the number of immobilized thiolated aptamers (Hassani et al., 2018a).

**FIGURE 2:**
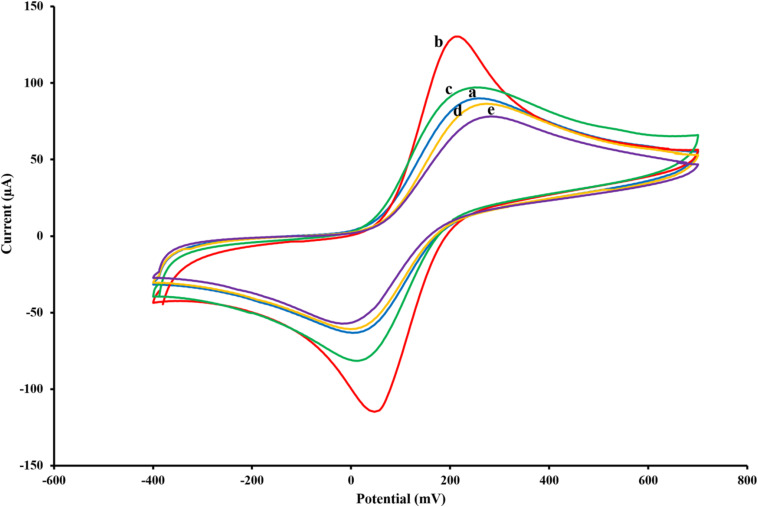
Cyclic voltammograms of the untreated SPCE **(A)**, AuNPs/SPCE **(B)**, aptamer/AuNPs/SPCE **(C)**, MCH/aptamer/AuNPs/SPCE **(D)** and BPA (100 pM)/MCH/aptamer/AuNPs/SPCE **(E)** in 0.1 M PBS (pH 7.5) containing [Fe(CN)_6_]^3–/4–^/KCl (5 mM/0.1 M) electrolyte solution.

The activation process by CV was carried out in H_2_SO_4_ solution (0.5 M) with potential ranging between −0.1 and 1.3 V and a scan rate of 0.1 V/s to obtain repetitive and fixed voltammograms. Recent studies suggest that the elimination of the oxide layer and the introduction of the highly active sites on the electrode surface after activation may lead to a significant improvement in electrochemical performance (Wan et al., 2015; Nazari et al., 2019). Characterization of the SPCE was conducted by CV (−0.4 to +0.7 V) with scan rates of 0.1 V/s in 0.1 M PBS (pH 7.5) containing [Fe(CN)_6_]^3–/4–^/KCl (5 mM/0.1 M) electrolyte solution as the electrochemical redox probe. Following activation of the electrode, deposition extent, and size of AuNPs coated on the SPCE could be significantly affected by the time of deposition (Arduini et al., 2016). To achieve the maximum electrochemical responses, the deposition time of AuNPs was fixed at 200 s, and CV measurements were subsequently performed with AuNPs modified and solely activated SPCEs in the solution of 0.1 M PBS (pH 7.5) containing [Fe(CN)_6_]^3–/4–^/KCl (5 mM/0.1 M; Figure 2).

### Label-Free Aptasensor BPA/MCH/Thiolated Aptamer/AuNPs/SPCEs Characterization

To monitor the electrochemical characteristics of the various modifications applied to the fabricated BPA-aptasensor, the electrochemical activity of the modified layers was analyzed using CV and EIS techniques. The entire experiments were performed in 0.1 M PBS (pH 7.5) containing 5 mM [Fe(CN)_6_]^3–/4–^ and 0.1 M KCl (Maghsoudi et al., 2020). CV measurements were recorded by sweeping the potential from −0.4 to +0.7 V with a scan rate of 0.1 V/s. Figure 2 shows the redox probe CV scans of the bare SPCE (curve a), AuNPs/SPCE (curve b), aptamer/Au NPs/SPCE (curve c), MCH/aptamer/AuNPs/SPCE (curve d) and 100 pM BPA/aptamer/AuNPs/SPCE (curve e). To reveal the EC aptasensor fabrication process, CV and EIS techniques were employed. The redox probe’s functional properties were explored in various stages, with different current response values and peak-to-peak separations (Δ*E*_p_ = *E*_pa_–*E*_pc_). The bare carbon SPE showed an acceptable reversible peak, which could be ascribed to the swift heterogeneous charge transfer features. Certain properties of [Fe(CN)_6_]^3–/4–^ were evaluated via redox peak separation and calculating Δ*E*_p_ of the cathodic and anodic waves and current response. The CV results of the redox probe at the SPCE indicated a pair of reversible reduction/oxidation peaks with Δ*E*_p_ of 253.90 mV (Figure 2, curve a). After modification of SPCE with AuNPs, the peak current experienced a great surge, and Δ*E*_p_ was dramatically dropped to 164.04 mV which could be attributed to the fact that AuNPs could significantly increase the conductivity of the electrode surface to facilitate the electrocatalytic activity and electron-transfer (Figure 2, curve b). When the thiolated aptamer was modified on the AuNPs/SPCE surface by a self-assembly process, a remarkable decline in redox peak current and slight enhancement in Δ*E*_p_ (241.46 mV) was observed (Figure 2, curve c). This could be associated with the repulsive steric hindrance of the negatively charged phosphate groups of the aptamer hampering the electrolyte transfer. According to Figure 2, curve d, incorporation of MCH molecules on to the modified surface of the electrode led to a considerable decrease in the redox peak current and a parallel increase in the peak separation (Δ*E*_p_ 286.11 mV) (Florea et al., 2013). Finally, the peak current value underwent a noticeable decrease after the reaction of aptasensors with 100 pM BPA analyte for 40 min, and the peak potential separation rose correspondingly (Δ*E*_p_ 298.32 mV) (Figure 2, curve e).

These observations are in agreement with previous reports, which offered a tentative explanation for the current changes; the formation of the immobilized aptamer-target complex may result in a secondary structure like a stem-loop, which could interfere with the electrochemical properties of the aptasensor (Hassani et al., 2018a).

### Optimization of the Analytical Parameters for BPA Determination

To achieve the best outcomes for the BPA assay, some essential experimental conditions including AuNPs deposition time, aptamer concentration, self-assembly time, MCH concentration, incubation time, pH value, and finally, analyte incubation time were optimized. As shown in Supplementary Figure S1, different deposition times of AuNPs were assessed using CV. The current response value sharply increased from 50 to 200 s and then began to fall gradually. Therefore, the electrodeposition time of 200 s was found to be optimal to achieve the maximum current response (Supplementary Figure S1, inset). The aptamer concentration is another critical factor in the fabrication of the aptasensor to attain the maximum sensitivity to the analyte. For this purpose, 10 μL of different aptamer concentrations from 0.1 to 5 μM were reacted with the aptasensor and electrochemical properties were recorded by the DPV technique to reach the maximum response value to BPA. As depicted in Supplementary Figure S2A, the DPV peak current response was enhanced by increasing the aptamer concentration and then tended to constant values after 1 μM, suggesting that saturation of the active sites of modified electrode surface by thiolated aptamer occurred. As the excess BPA-aptamer concentration could block the electron transportation process, a higher concentration of thiolated aptamer decreased the current response; therefore, 1 μM was selected as the optimal aptamer concentration for further assays. The effect of time on the self-assembly process was evaluated with different incubation times within the range of 4–24 h (Supplementary Figure S2B). The incubation time of the thiolated BPA aptamer on the modified SPCE surface was optimized as it is an important variable affecting the efficiency of the aptasensor. The peak current response rose steeply as incubation time increased between 4 and 16 h and then reached a plateau after 16 h, confirming that aptamer saturation on the electrode surface occurred. It seemed that exceeding the time to more than 16 h resulted in the saturation of the active binding sites.

Moreover, a more extended incubation period led to the aggregation of aptamers on the electrode surface. Hence, we chose 16 h as the optimum time for a self-assembly reaction. As stated earlier, MCH was used for deactivation and blocking of free active sites and untreated regions of the electrode surface. Optimization of the concentration in the range between 0.5 and 10 μM and incubation time ranging from 20 to 60 min of MCH are shown in Supplementary Figures S2C,D, respectively. The results demonstrated notable increases in the signal current of DPV scans in MCH concentration ranging from 0.5 to 2 μM and in a span of 20 to 30 min incubation time. The current response reached its steady-state after these data points. Accordingly, 2 μM and 30 min were selected as optimal MCH conditions. The effect of time required for BPA analysis was evaluated in a period between 10 and 60 min. As shown in Supplementary Figure S2E, following the insertion of 100 pM BPA on the modified aptasensor surface the output current was amplified rapidly as the incubation time increased and reached the maximum level at 40 min time point, and then remained approximately constant which could be attributed to the electrode surface saturation. For this reason, the incubation time of 40 min was set for subsequent analyses. As shown in Supplementary Figure S2F, the effect of pH values (5.0–8.0) of the buffer solution on signal current response is presented. It is noteworthy that the pH value of the buffer solution is regarded as one of the critical contributing factors in the optimum aptamer performance. The maximum peak current response was achieved at a pH value of 7.5, while in the pH ranging from 7.5 to 8.0, a dramatic decrease was observed in the peak current.

Considering the pKa of BPA (pKa = 9.73 as a weak acid), it indicates that most of BPA species is in non-ionic form, and there is a possibility at higher and lower pHs BPA changes to an ionic form. Then, the data in the graph point out that neutral form adsorbed on the modified surface of the electrode better than the ionic forms of BPA. Consequently, the pH 7.5 was selected as the optimal working pH for the experimental conditions (Fernández et al., 2006; Fan et al., 2012; Han et al., 2015).

### EIS Spectra of the Modified Electrode

**FIGURE 3:**
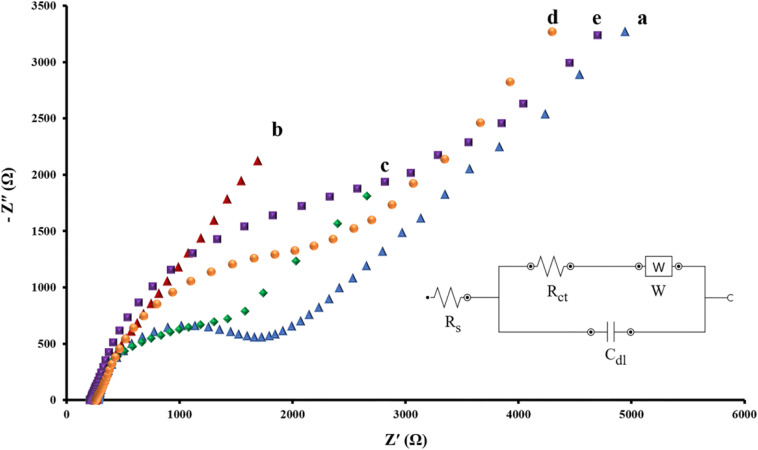
Nyquist spectra of EIS data for intact SPCE **(A)**, AuNPs/SPCE **(B)**, aptamer/AuNPs/SPCE **(C)**, MCH/aptamer/AuNPs/SPCE **(D)** and BPA (100 pM)/MCH/aptamer/AuNPs/SPCE **(E)** in 0.1 M PBS (pH 7.4) containing [Fe(CN)_6_]^3–/4–^/KCl (5 mM/0.1 M) electrolyte solution. The Randles equivalent circuit was employed to model the electrochemical impedance data, (*R*_s_: solution resistance; *C*_dl_: double layer capacitance; *R*_ct_: charge transfer resistance, *W*: Warburg impedance).

The EIS is regarded as one of the most practical tools to assess real conditions of aptasensors in all fabrication steps. Impedance spectrum (Nyquist diagram) consists of two major compartments: a semicircle segment at high-frequency regions correlating to the charge-transfer resistance and a linear segment in the low-frequency range corresponding to the diffusion process. The diameter of the semicircle stands for the charge-transfer resistance (*R*_ct_) at the electrode surface and the straight-line accounting for the diffusion resistance (Han et al., 2016). The Randles equivalent circuit was employed to model the electrochemical impedance data (Figures 3, inset).

EIS data was recorded at a frequency ranging between 0.01 and 100 kHz and an AC voltage amplitude of 0.01 V, superimposed on a DC (direct current) of +0.13 V. Figure 3 represents the Nyquist plots of impedance data of each modification step of the aptasensor in 0.1 M PBS (pH 7.5) containing [Fe(CN)_6_]^3–/4–^/KCl (5 mM/0.1 M) electrolyte solution.

According to Figure 3, the curve a corresponds to the impedance of the bare SPCE indicates the *R*_ct_ value of about 1.723 kΩ at the electrode surface. The electrodeposition of AuNPs on the working electrodes of bare SPCE resulted in a sharp reduction of the *R*_ct_ level to 0.282 kΩ (Figure 3, curve b). As previously confirmed by CV, conductivity was considerably enhanced due to the expansion of the active surface area.

Subsequent immobilization of thiolated capture aptamer and MCH onto the AuNPs/SPCE caused the semicircle’s diameter of the Nyquist plot to increase significantly. As a result of the blockade of charge transfer between SPCE and the redox probe, the *R*_ct_ level rose from 1.302 kΩ (Figure 3, curve c) to 2.019 kΩ (Figure 3, curve d). Finally, when the immobilized aptamer attached to its target BPA (100 pM for 40 min incubation), substantial steric hindrance effect of the aptamer-BPA complex resulting in a noticeable rise in the *R*_ct_ value to 2.813 kΩ, demonstrated that the fabricated aptasensor interacted successfully with BPA (Figure 3, curve e) (Deiminiat et al., 2017).

### Analytical Performance of the Electrochemical Aptasensor

**FIGURE 4:**
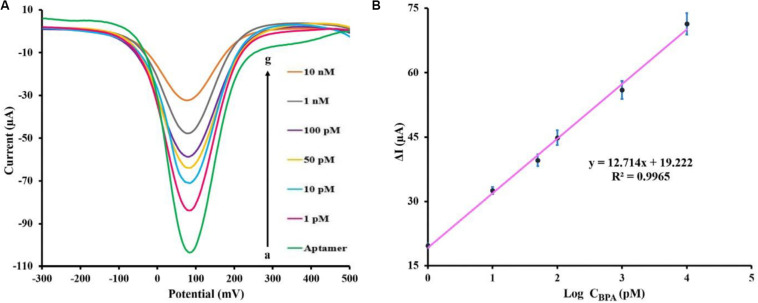
**(A)** DPV peaks of the fabricated aptasensor measuring various BPA concentrations [0, 1, 10, 50, 100 pM, 1 and 10 nM] in 0.1 M PBS (pH 7.5) containing [Fe(CN)_6_]^3–/4–^/KCl (5 mM/0.1 M) electrolyte solution. **(B)** BPA standard calibration curve in PBS (0.1 M), the error bars represent the standard deviation of three consecutive measurements.

DPV method was utilized to investigate the efficacy of the fabricated aptasensor in the quantitative analysis of BPA. The quantitative detection of BPA was carried out using DPV under the optimum experimental conditions (potential range +0.5 to −0.3 V, amplitude modulation of 0.05 V, and interval time 0.2 s). The aptasensor was incubated for 40 min in various concentrations of BPA prepared in the PBS 0.1 M (pH 7.5) and subsequently was detected in a solution of 5 mM [Fe(CN)_6_]^3–/4–^ containing 0.1 M KCl (pH 7.5). As shown in Figure 4A, the corresponding DPV peak current with increasing BPA concentrations showed a significant decrease. The relative peak current changes of the aptasensing platform were linear over the range of 1 pM to 10 nM BPA concentration. Figure 4B shows the performance of the aptasensor at different BPA concentrations by DPV. The limit of detection (LOD) of the aptasensor was 0.113 pM (based on the formula: LOD = 3Sb/S, where Sb and S represent the standard deviation of the blank and the slope of the calibration curve, respectively) using the linear regression equation ΔI (μA) = 12.714 log C_BPA_ (pM) + 19.222 and correlation coefficient of 0.9965.

Previously reported electrochemical methods for BPA detection are shown in Table 1. By comparison with other techniques, our designed aptasensor showed the least LOD and broader linear range. Therefore, this aptasensor exhibits remarkable potential for ultra-low-level measurements of BPA. When examined on five distinct modified SPCEs, the DPV method demonstrated that BPA (100 pM) was detected with an acceptable relative standard deviation (RSD) value of 3.6%, confirming that the test was reproducible under the experimental condition. The long-term stability of the fabricated aptasensor was investigated, measuring 100 pM BPA solution in a regular fashion via three SPCEs kept fortnightly at 4°C. During this period, the response value experienced less than 9.4% decline compared with the initial response, which was not statistically significant. This indicated that no considerable decomposition occurred, and fabricated biosensor remained perfectly stable in long-term storage.

**TABLE 1:** Comparison of BPA analytical performances of recent publications.

**Detection method**	**Biosensor configuration**	**Linear range**	**LOD**	**References**
Electrochemical	Laccases/Ag – ZnONPs/MWCNTs/SPCEs	0.5 – 2.99 μM	6.0 nM	Kunene et al., 2020
Colorimetric	Colloidal gold nanoparticles, BPA-specific 24-bp aptamer, and NaCl as an electrolyte	0.004 – 4380 nM	0.004 nM	Lee et al., 2019
Electrochemiluminescence	BSA/Aptamer/AuPs/NCDs@PEI – rGO/RuNSs/GCE	0.1 nM – 0.1 mM	33 pM	Liu et al., 2019
Electrochemical	BSA/Anti-BPA/AuNPs/MWCNTs/GCE	10 nM – 1 μM	8.7 nM	Huang et al., 2016
Optical-FRET	Aptamer-KGdF_4_:Tb^3+^ NPs/cDNA-AuNPs	2 – 400 nM	0.64 nM	Duan et al., 2015
Optical-SPR	Biotin-modified Ab (BPA)/Streptavidin-modified gold nanoparticles/SPR chip	0.04 nM – 400 μM	20.8 pM	Xue et al., 2019
Electrochemical	Aptamer/MWCNT-SiO2@Au nanocomposite/GCE	0.1 – 100 nM	10 pM	Razavipanah et al., 2019
Electrochemical	BSA/anti-BPA/AuNPs/AB-CS-Au/GCE	7.5 nM – 1 μM	6.4 nM	Li et al., 2017
Electrochemical	MCH/Thiolated label-free Aptamer/AuNPs/SPCE	1 pM – 10 nM	0.113 pM	Present study

*Abbreviations: LOD, limit of detection; SPCEs, screen printed carbon electrode; Ag-ZnONPs, silver doped zinc oxide nanoparticles; MWCNTs, multi-walled carbon nanotubes; GCE, glassy carbon electrode; RuNSs, Ru(bpy)_3_^2+^ nanosheets; AuPs, gold particles; NCDs@PEI, poly(ethylenimine) functionalized nitrogen-doped carbon quantum dots; rGO, reduced graphene oxide; AuNPs, gold nanoparticle; FRET, fluorescence resonance energy transfer; KGdF_4_:Tb^3+^ NPs, lanthanide-doped KGdF_4_ nanoparticles; AB-CS-Au, acetyleneblack-chitosan-gold composite; cDNA, complementary DNA; SPR, surface plasmon resonance; MCH, 6-mercapto-1-hexanol.*

### The Selectivity of the Aptasensor

**FIGURE 5:**
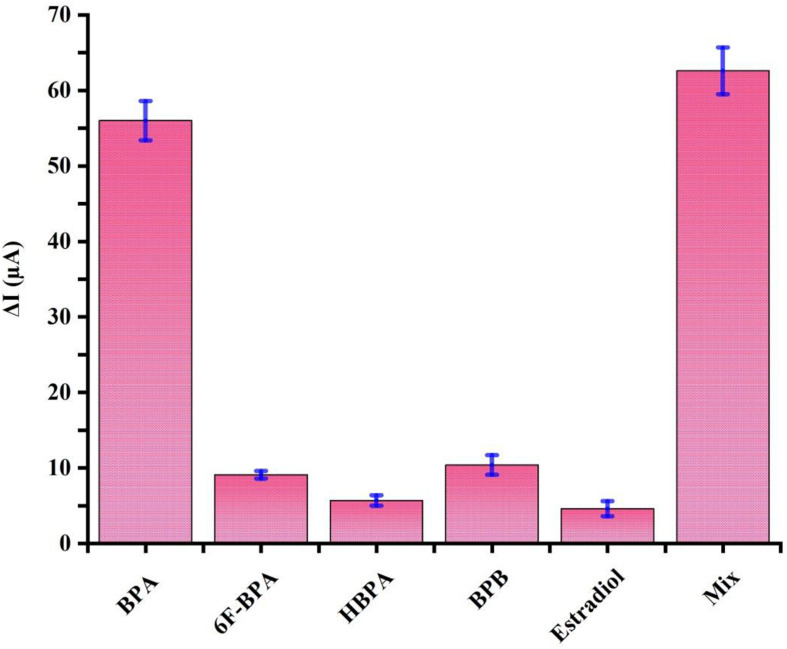
Specificity evaluation of the proposed aptasensor for 100 pM BPA by comparing its DPV signals to 1 nM of well-known interfering agents, including 6F-BPA, HBPA, BPB, and estradiol and the mixture. Error bars represent standard deviations of three sets of parallel experiments.

Specificity plays a significant role in biosensing platforms to detect and quantify the target substance accurately in real samples. To examine the selectivity of the aptasensor, similarly structured compounds, including BPB, HBPA, 6F-BPA, and estradiol at a concentration of 1 nM were prepared as interfering species in a mixture with 100 pM BPA. As illustrated in Figure 5, the DPV signals of these chemicals were considered negligible compared to BPA electrochemical signal. Moreover, the response signals recorded in the mixture of all the chemicals and the solution containing the sole target BPA were virtually the same. These results confirmed that there was an insignificant response for BPA analogs, and the proposed electrochemical aptasensor could be exploited to determine BPA with strong selectivity.

### Real Sample Analysis

**FIGURE 6:**
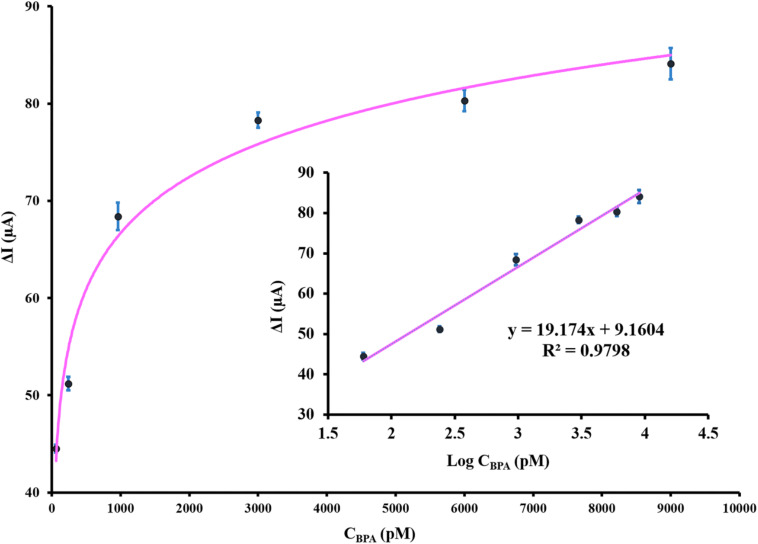
The calibration curve of the designed aptasensor after incubation with various spiked concentrations of BPA in plasma samples. The inset depicts the linear relationship between DPV peak current response and BPA concentration. The error bars indicate the standard deviation of triplicate experiments.

To evaluate the capability of the aptasensor in BPA detection in real samples, five different spiked concentrations of BPA in the plasma samples of male *Wistar* rats (untreated group) were diluted five times with 0.1 M PBS (pH 7.5) and analyzed using the proposed aptasensor. The results (Table 2) revealed that the recovery value was in the range of 93.8 – 97.3% and RSD varied from 2.46 to 4.53%. ELISA method was performed to confirm the reliability of the proposed aptasensor and its recovery and RSD were ranging from 92.4 to 96.3 and 2.56 to 4.11%, respectively. The analytical results of the fabricated aptasensor are well compatible with those obtained by the ELISA method, indicating the trustworthiness and reasonable accuracy of the application of aptasensor in real samples. The linear regression equation of ΔI (μA) = 19.174 log C_BPA_ (pM) + 9.1604 and correlation coefficient of 0.979 were obtained from the real samples and are presented in Figure 6. These results underlined the enormous potential of the developed label-free aptamer to detect precisely ultra-trace amounts of BPA in clinical samples.

**TABLE 2:** Determination of BPA in the plasma of male Wistar rats using the proposed method and ELISA technique (*n* = 5).

		**Aptasensor**			**ELISA**		
**Samples**	**Spiked (pM)**	**Found (pM)**	**Recovery (%)**	**RSD (%)**	**Found (pM)**	**Recovery (%)**	**RSD (%)**
1	100	93.80	93.8	2.71	92.4	92.4	3.42
2	500	482.0	96.4	2.46	475.1	95.0	4.11
3	1000	945.2	94.5	4.12	963.8	96.3	2.56
4	3000	2873.1	95.7	3.28	2835.2	94.5	3.97
5	8000	7786.5	97.3	4.53	7691.2	96.1	3.91

## Conclusion

In this project, a novel and facile signal off electrochemical aptasensing strategy were introduced based on SPCE modification with AuNPs to facilitate charge transfer and conjugation to thiolated aptamers for effective BPA sensing. Due to the high affinity for BPA molecules, the proposed aptasensor offered exquisite selectivity and sensitivity for BPA determination in real samples. Owing to the intrinsic features of electrochemical transducers such as adaptability, miniaturization, simplicity, minimum sample requirement, and swiftness, biosensing techniques can be efficient. It can be regarded as a significant future detection device for food and environmental contaminants. Under the optimum conditions, our EC aptasensor provides a low detection limit of 0.113 pM and a linearity index between 1 pM and 10 nM for BPA. Fortunately, the present method is very much compatible with the ELISA reference technique and confirms the reliability and accuracy of the application of aptasensor in real samples.

The outstanding performance of the aptasensor is associated with superb aptamer-based techniques in comparison to antibody-based techniques and nanoparticle-based signal amplification strategy. Implementation of screen printing technology has exceptional merits, including reproducibility, portability, the least interaction with other components in real samples, and a higher probability of commercialization. Development and application of EC aptasensor for determination of BPA in our study would be a credible and economical alternative to the conventional analyzing methods, as precise quantification of BPA as one of the most prevalent EDCs is of great importance. Biosensing strategies, particularly aptasensors, would be readily credible for the detection of other environmental contaminants to improve food safety and mitigate environmental pollution.

## Data Availability Statement

The original contributions presented in the study are included in the article/Supplementary Material, further inquiries can be directed to the corresponding author.

## Ethics Statement

The animal study was reviewed and approved by Tehran University of Medical Sciences Ethics Committee.

## Author Contributions

All authors made considerable contributions to design, the data gathering, and analysis, equally participated in drafting the manuscript or revision, and agreed to be responsible for all aspects of the project.

## Conflict of Interest

The authors declare that the research was conducted in the absence of any commercial or financial relationships that could be construed as a potential conflict of interest.

## Supplementary Material

FIGURE S1 The CVs of SPCEs at different electrodeposition time of AuNPs on the electrode surface.

FIGURE S2 Optimization of the experimental parameters: effects of **(A)** aptamer concentration, **(B)** self-assembly time, **(C)** MCH concentration, **(D)** MCH incubation time, **(E)** BPA incubation time, and **(F)** pH values.
