# Supplementary material for: High-Performance Voltammetric Aptasensing Platform for Ultrasensitive Detection of Bisphenol A as an Environmental Pollutant
Source: Front Bioeng Biotechnol. 2020 Sep 4;8:574846. doi: 10.3389/fbioe.2020.574846 (PMC7498542; doi:10.3389/fbioe.2020.574846)
Supplement: FIGURE S1 — The CVs of SPCEs at different electrodeposition time of AuNPs on the electrode surface. [file Image_1.pdf]

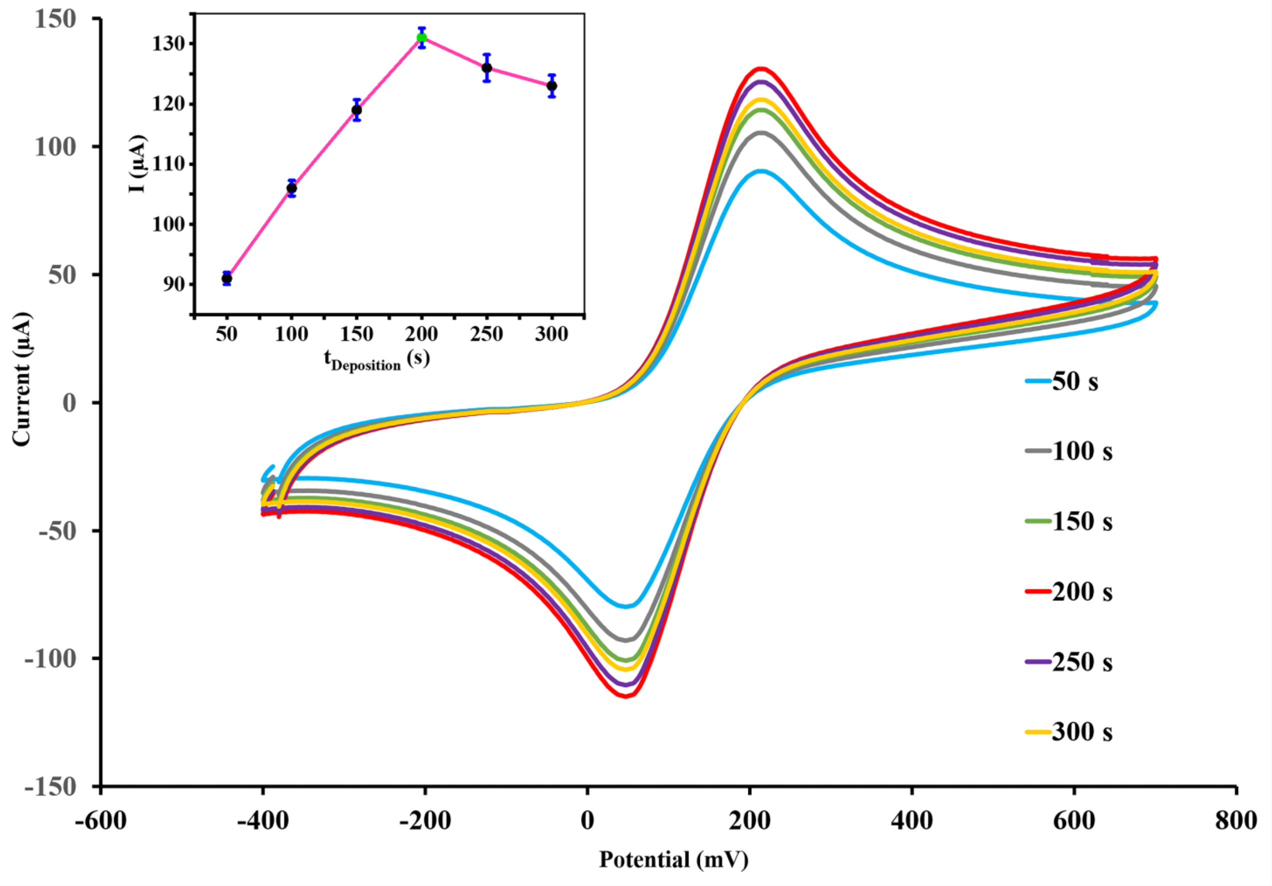

Figure S1. The CVs of SPCEs at different electrodeposition time of AuNPs on the electrode surface.
