# Supplementary material for: High-Performance Voltammetric Aptasensing Platform for Ultrasensitive Detection of Bisphenol A as an Environmental Pollutant
Source: Front Bioeng Biotechnol. 2020 Sep 4;8:574846. doi: 10.3389/fbioe.2020.574846 (PMC7498542; doi:10.3389/fbioe.2020.574846)
Supplement: FIGURE S2 — Optimization of the experimental parameters: effects of (A) aptamer concentration, (B) self-assembly time, (C) MCH concentration, (D) MCH incubation time, (E) BPA incubation time, and (F) pH values. [file Image_2.pdf]

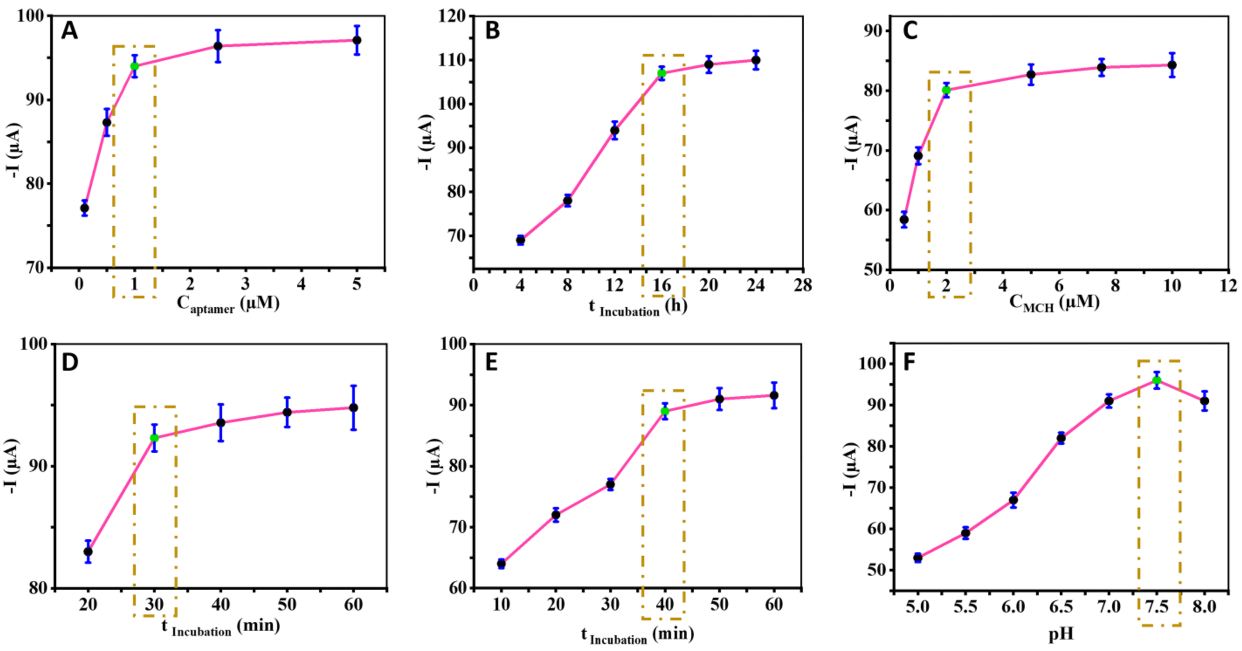


**Figure S2.** Optimization of the experimental parameters: effects of (A) aptamer concentration, (B) self-assembly time, (C) MCH concentration, (D) MCH incubation time, (E) BPA incubation time, and (F) pH values.
